# Brain-derived neurotrophic factor promotes immune reconstitution following radiation injury via activation of bone marrow mesenchymal stem cells

**DOI:** 10.1371/journal.pone.0259042

**Published:** 2021-10-25

**Authors:** Guru Prasad Sharma, Anne C. Frei, Jayashree Narayanan, Tracy Gasperetti, Dana Veley, Asma Amjad, Katherine Albano, Brian L. Fish, Heather A. Himburg

**Affiliations:** 1 Department of Radiation Oncology, Medical College of Wisconsin, Milwaukee, Wisconsin, United States of America; 2 Cancer Center, Medical College of Wisconsin, Milwaukee, Wisconsin, United States of America; Children’s Hospital Boston, UNITED STATES

## Abstract

Brain-derived neurotrophic factor (BDNF) is a member of the nerve growth factor family which has been extensively studied for its roles in neural development, long-term memory, brain injury, and neurodegenerative diseases. BDNF signaling through tropomyosin receptor kinase B (TrkB) stimulates neuronal cell survival. For this reason, small molecule TrkB agonists are under pre-clinical develoment for the treatment of a range of neurodegenerative diseases and injuries. Our laboratory recently reported BDNF is secreted by pro-regenerative endothelial progenitor cells (EPCs) which support hematopoietic reconstitution following total body irradiation (TBI). Here we report BDNF-TrkB signaling plays a novel regenerative role in bone marrow and thymic regeneration following radiation injury. Exogenous administration of BDNF or TrkB agonist 7,8-dihydroxyflavone (7,8-DHF) following myelosuppressive radiation injury promoted faster recovery of mature blood cells and hematopoietic stem cells capable of multi-lineage reconstitution. BDNF promotes hematopoietic regeneration via activation of PDGFRα^+^ bone marrow mesenchymal stem cells (MSCs) which increase secretion of hematopoietic cytokines interleukin 6 (IL-6) and leukemia inhibitory factor (LIF) in response to TrkB activation. These data suggest pharmacologic activation of the BDNF pathway with either BDNF or 7,8-DHF may be beneficial for treatment of radiation or chemotherapy induced myelosuppression.

## Introduction

Bone marrow vascular and perivascular stromal cells are known to regulate hematopoietic regeneration following radiation injury [1]. Recently, our laboratory demonstrated therapeutic administration of cord blood-derived endothelial progenitor cells (EPCs) rescued bone marrow function following high doses (5–8 Gy) of total body irradiation (TBI) in mice [2]. A cytokine screen was performed to identify candidate pro-regenerative factors secreted by the infused EPCs and among the factors identified was the neurotrophic growth factor brain-derived neurotrophic factor (BDNF) [2]. In this current manuscript, we establish a novel role for BDNF-TrkB signaling in the regenerating bone marrow.

BDNF has been well characterized as a growth factor for neurons where it has been shown to promote neural cell survival and regeneration through activation of its most high affinity receptor, tropomyosin receptor kinase B (TrkB). Systemic BDNF administration promotes neurogenesis following ischemic stroke [3, 4]. The small molecule agonist of TrkB 7,8-dihydroxyflavone (7,8-DHF) has been developed and tested pre-clinically for the treatment of a range of neurodegenerative diseases and injuries including: traumatic brain injury, Parkinson’s disease, Alzheimer’s disease, Huntington’s disease, and stroke [5–10]. 7,8-DHF is a naturally occurring flavone which was developed therapeutically as a more potent, longer-lasting alternative to BDNF for use in clinical trials of neurodegenerative disorders [7]. 7,8-DHF binds and activates TrkB in a similar manner as BDNF; however, 7,8-DHF has a significantly longer half-life (134 min vs. <10 min) and does not initiate TrkB degradation [7, 10, 11]. In addition to its regenerative roles in neuronal cell biology, work from the Hempstead lab has shown BDNF also regulates vascular development, angiogenesis, and response to vascular injury [12–15]. Of note, they observed BDNF administration induced mobilization of bone marrow hematopoietic progenitors to sites of ischemic injury [14]. Recently, BDNF-mediated activation of lung mesenchymal cells has been shown to promote alveolar regeneration following lung injury [16].

Expression of neurotrophins and their receptors in human bone marrow were first noted by Labuoyrie et al. in 1999 [17]. Since this time, human bone marrow and cord blood CD34+ cells have been shown to express the neurotrophin receptors TrkA, TrkB, and TrkC [18, 19]. Moreover, Schuhmann et al. demonstrated a fundamental role for BDNF in B-cell development as mice with a genetic deletion of BDNF have an impairment in B-lymphocyte maturation [20]. BDNF-TrkB signaling is also thought to play a role in T-lymphocyte development as loss of TrkB function induced apoptotic death of cortical thymic lymphocytes [21]. However, there is no established role for BDNF-TrKB signaling in the bone marrow or thymus following myeloablative injury.

## Materials and methods

### Animals

All experimental procedures were carried out according to the National Institutes of Health (NIH) Guide for the Care and Use of Laboratory Animals and approved by the Institutional Animal Care and Use Committee (IACUC) of the Medical College of Wisconsin. Equal numbers of male and female C57BL/6 or B6SJL mice 4–10 weeks were purchased from Jackson labs. For imaging studies, BDNF-TdTomato reporter mice were generated by crossing BDNF-Cre (Jackson labs strain 030189 with Rosa26 TdTomato (Jackson labs strain 007914) mice [22, 23]. Mice received total body irradiation (TBI) to a dose of 5 or 9 Gy. Irradiations were performed between 8–10 am on unanesthetized mice using a SmART Precision X-ray instrument (225 kVP; 10 mA; half value layer (HVL) 0.89 mm Cu) with a dose rate of 107.7 cGy/min.

### In vivo administration of experimental therapeutics

Recombinant human BDNF (100% sequence homology with mouse BDNF) and the small molecule agonist 7,8-dihydroxyflavone (7,8-DHF) were purchased from Biotechne (Minneapolis,MN). Mice were dosed starting at 24 hours after TBI and drug treatment was administered every other day through day 10. BDNF was reconstituted in sterile PBS and administered subcutaneously at 0.5 mg/kg [3, 4]. 7,8-DHF was administered subcutaneously at 1 mg/kg in PBS with 0.1% DMSO [24].

### Cell isolation

For isolation of hematopoietic cell populations, bone marrow from individual femurs was flushed using an insulin syringe into 5 ml of sterile PBS + 10% FBS. Cell pellets were lysed with ACK buffer and filtered. Prior to FACS sorting stem cell populations, column purification was performed to enrich for lineage negative cells (Miltenyi lineage cell depletion kit, 130-090-858). For analysis of thymocyte populations, thymi were dissected, cleaned of excess fat and connective tissue, and triturated by passing through 40μm strainer. Viable cells were enumerated by trypan blue exclusion on a Countess cell counter. For isolation of endothelial and stromal cell populations, long bones were harvested and crushed with a mortar and pestle in PBS. The crushed bone product was then digested at 37 C for 10 minutes in a collagenase/dispase buffer (2.5 mg/ml Collagenase A (Sigma, C2139-500MG), 1 unit/ml Dispase II (Stemcell Technologies, 07913). Following digestion, bones were rinsed in ice-cold complete media (IMDM + 10% FBS), filtered, and then column depleted of lineage committed hematopoietic cells (Miltenyi Biotec, Auburn, CA).

### Flow cytometry sorting and analysis

FACS sorting was performed at the Versiti Blood Research Institute Flow Cytometry Shared Resource. Flow cytometric analysis was performed using a Miltenyi MACSQuant 10 instrument.

The lineage-Sca-1+c-Kit+ (LSK) expressing hematopoietic stem and progenitor cell population were labeled with PE anti-mouse CD117 (c-Kit, Biolegend 105808), APC/Cyanine7 anti-mouse Ly-6A/E (Sca-1, Biolegend 108126), and Pacific Blue anti-mouse Lineage Cocktail (Biolegend 133310). Common myeloid progenitor (CMP: lineage-c-Kit+ Sca-1-CD34+CD16/32-) and common lymphoid progenitor (CLP: LSK IL-7Rα+) subsets were discriminated using by the addition of the following antibodies to the LSK panel: Brilliant Violet 510 anti-mouse CD16/32 (Biolegend 101333), PE/Cyanine7 anti-mouse CD34 (128618), APC anti-mouse CD127 (IL-7Rα, Biolegend 135012). Discrimination of donor B6.SJL versus recipient C57BL/6 blood cell lineages was performed using: FITC anti-mouse CD45.1 (Biolegend 110706), PE anti-mouse CD45.2 (Biolegend 109808), Pacific Blue anti-mouse CD3 (Biolegend 155612), APC/Cyanine7 anti-mouse B220 (Biolegend 103224), Brilliant Violet 510 anti-mouse TER-119 (Biolegend 116237), PE-Cy7 Cd11b (Biolegend 101216) and APC anti-mouse Gr-1 (Biolegend 108412) antibodies. Thymocytes were analyzed for double negative (CD4- CD8-) and double positive (CD4+ CD8+) populations using CD4-VioBlue (Miltenyi 130-118-568), CD8a-PE/VIO770 (130-118-946), CD44-APC (Miltenyi, 130-119-121), CD25 PE (130-120-696).

### Endothelial and stromal cell analysis

For FACS-sorting CD31 and PDGFRα populations, the following antibodies were used: FITC anti-mouse CD45 (Biolegend 103108), APC anti-mouse CD31 (Miltenyi 130-123-813), and PE-Vio770 CD140a (Miltenyi, 130-105-116). For analysis of endothelial cell death and mitochondrial ROS generation, the following antibodies were used: BV421 anti-mouse CD45 (Biolegend 103133), AF647 anti-mouse VE-Cadherin (Biolegend 138006). For these studies, mice were intravenously labeled with VE-Cadherin antibody (25 ug/mouse) via IV injection. Fifteen minutes following injection, mice were sacrificed, long bones were harvested and digested as described above. Endothelial cells were identified as the CD45- 7AAD- VE-Cadherin+ cell population. To discriminate apoptotic cells within this population, the CellEvent™ Caspase-3/7 Green Flow Cytometry Assay Kit (ThermoFisher, C10427) was used per manufacturer’s instructions. Mitochondrial ROS was measured using MitoSOX Red (ThermFisher, M36008).

### Enzyme‐linked immunosorbent assay (ELISA)

Bone marrow supernatants were obtained by flushing both femurs from a single mouse into a total volume of 400 ul cold PBS. Cells and debris were pelleted by centrifugation and the remaining supernatant was assayed at 1X for mature BDNF and pro-BDNF using the Mature BDNF/proBDNF Combo Rapid ELISA Kit purchased from Biosensis (BEK-2211/2217) per manufacturer’s instructions. Absorbance at 450 nm was measured on a Biotek plate reader.

### Complete blood counts (CBCs)

Complete blood counts were determined using a Heska Element 5 Veterinary Hematology Analyzer.

### Competitive transplantation

Congenic transplantation studies were performed using 500,000 whole bone marrow (WBM) cells isolated from B6.SJL at day 7 following 5 Gy TBI. Donor B6.SJL cells were transplanted into 9 Gy irradiated C57BL/6 mice along with a host competitor cell dose of 200,000 C57BL/6 WBM cells.

### Evans Blue Dye permeability assay

Evans Blue Dye (EBD) extravasation in the bone marrow space was assessed as previously described [25, 26]. Briefly, at 72 hours post-radiation and treatment, mice were injected intravenously with 200 ul of a 0.5% solution of EBD. Mice were euthanized 15 minutes post-injection and the bone marrow supernatant was collected. EBD concentration was determined by measuring absorbance on a spectrophotometer at 610 nm.

### Immunofluorescent imaging

Mice were intravenously labeled with VE-Cadherin antibody (25 ug/mouse, Biolegend) via IV injection. Fifteen minutes following injection, mice were sacrificed and long bones were harvested. Whole femurs from VE-cadherin-labeled mice were fixed overnight in 4% paraformaldehyde, decalcified for 72 hours in EDTA/PBS, incubated for 2 hours in 20% sucrose, and then embedded in OCT medium. Femurs were sectioned at 8 um. Sections were re-hydrated in PBS and mounted in ProLong Gold Antifade with DAPI. Slides were imaged at 20X using an EVOS M500 imaging system.

### Cytokine assay

Human primary bone marrow mesenchymal stromal cells were purchased from Stem Cell Technologies (70022) and cultured to confluence in MesenCult™ Proliferation Kit (Human; Catalog #05411). After reaching confluence the media was replaced with serum free basal medium with or without 100 ng/ml BDNF. The cells were irradiated in vitro with 8 Gy. At 24 hours, the cell culture supernatant was collected and concentrated 2-fold. A human cytokine 71-plex Discovery assay was performed by Eve Technologies. Samples that were out of the detection range were excluded from analysis.

### Statistical analysis

Values are reported as mean ± SEM, unless stated otherwise. All comparisons were performed using GraphPad Prism 9.0. All data were checked for normal distribution and similar variance between groups. Data are derived from multiple independent experiments from distinct mice or cell culture plates. Sample size for in vitro studies was chosen based on observed effect sizes and standard errors from prior studies.

## Results

### Characterization of bone marrow BDNF and TrkB expression

**Fig 1:**
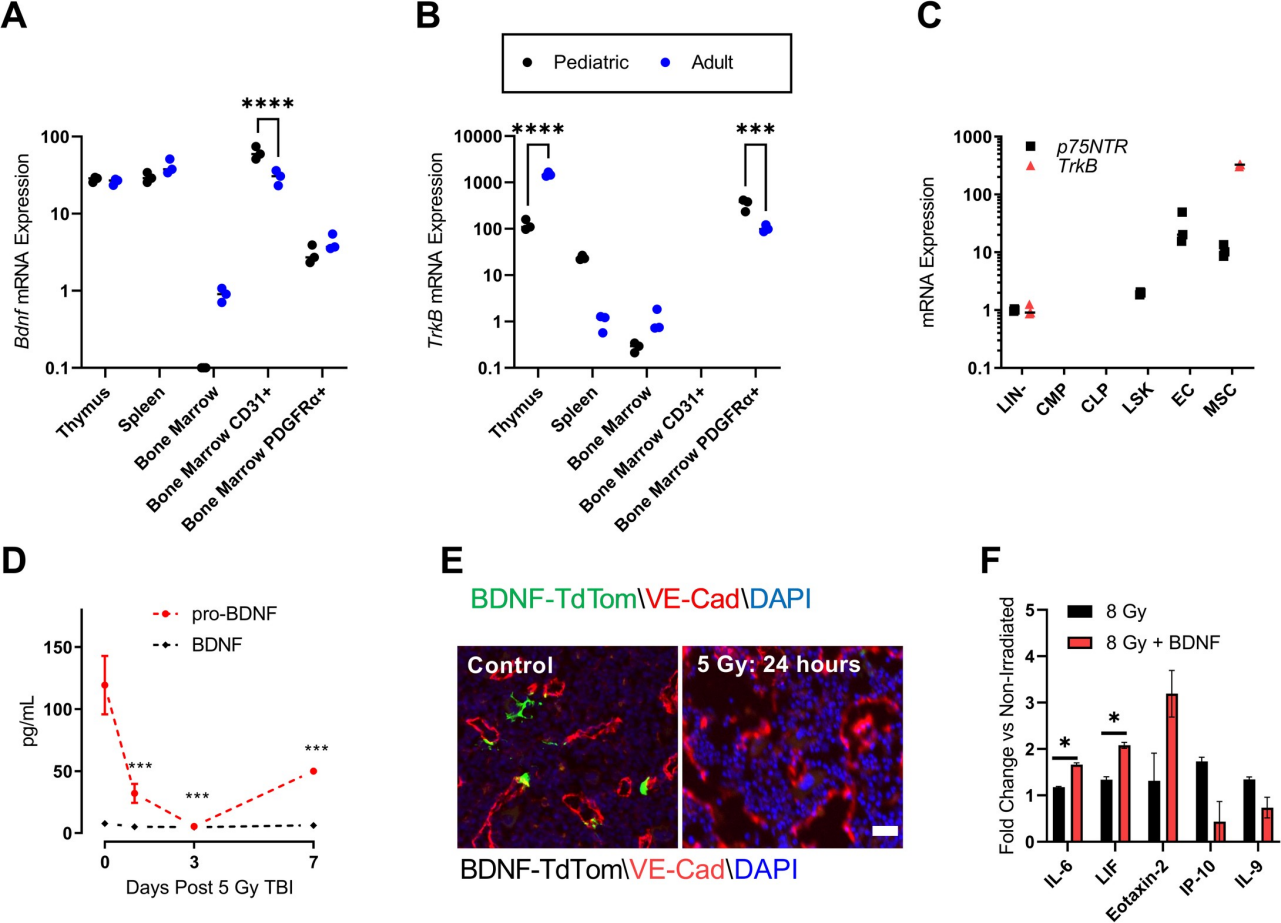
BDNF and TrkB expression in the bone marrow niche. (A) *Bdnf* mRNA expression in the thymus, spleen, bone marrow CD31+ endothelial cells (ECs), and bone marrow PDGFRa+ mesenchymal stromal cells (MSCs). (B) *TrkB* mRNA expression in the same populations. (C) *p75NTR (NGFR)* and *TrkB* mRNA expression in sorted hematopoietic stem and progenitor cell populations. Data are based on cells sorted from three mice per group. (D) ELISA analysis of BDNF and pro-BDNF protein concentration in the bone marrow supernatant following 5 Gy total body irradiation (TBI). n = 3 mice per timepoint. (E) Representative distribution of BDNF (green) in longitudinal femur sections labeled with VE-cadherin (red) and DAPI (blue) of non-irradiated control (left) and at 24 hours following 5 Gy TBI. Scale bar = 50 μm. (F) Fold change in MSC cell culture supernatant levels of cytokines IL-6, LIF, Eotaxin-2, IP-10, and IL-9 following either 8 Gy (black bar) or 8 Gy + 100 ng/ml BDNF (red bar). Fold change is relative to non-irradiated control MSCs. n = 3 replicates per treatment. Significance was determined using multiple t-tests with Bonferroni correction. Shown are mean values +/- SEM. *P < .05, ***P < .001, ****P < .0001.

*Bdnf* and *TrkB* mRNA levels were measured in the thymus, spleen, and bone marrow of pediatric (4 week old) and adult (12 week old) C57BL/6 mice (Fig 1A and 1B). Consistent with prior reports [20, 21, 27], *Bdnf* was highly expressed in the thymus and spleen. In the bone marrow, *Bdnf* is expressed most highly by the CD31^+^ endothelial cell fraction; however, within this fraction *Bdnf* transcript levels significantly decrease between 4 and 12 weeks of age. TrkB is a tyrosine kinase receptor with high affinity for BDNF. Prior studies have shown *TrkB* is expressed in both stromal and hematopoietic cell populations [17, 18]. Here we observed high expression of *TrkB* in the thymus and within PDGFRα^+^ expressing mesenchymal stem cells (MSCs). Interestingly, mRNA levels of *TrkB* rose in the thymus but declined in the BM PDGFRα^+^ with age. It is also worth noting that *TrkB* was not detectable within the CD31^+^ endothelial cell fraction. We then performed an additional analysis to determine if *TrkB* or the low affinity neurotrophin receptor *p75NTR* (NGFR) were expressed in FACS sorted bone marrow hematopoietic progenitor cell populations (Fig 1C). Neither *TrkB* or *p75NTR* transcript was detectable in the common myeloid progenitor (CMP) or common lymphoid progenitor (CLP) subsets. Within the stem cell-enriched lineage negative sca-1+ c-kit+ (LSK) fraction, only *p75NTR* was detectable at low levels.

We assessed bone marrow protein levels of BDNF and BDNF precursor pro-BDNF by ELISA before and following 5 Gy TBI (Fig 1D). Although levels of BDNF were not changed following radiation, we observed a significant decrease in BDNF precursor pro-BDNF at days 1–14 post injury. We then visualized BDNF expression in the bone marrow using a BDNF-TdTomato reporter mouse (BDNF-Cre mouse X Rosa26 TdTomato mouse [22, 23]). The vasculature was labeled by intravenous infusion of anti-VE-Cadherin antibody [2]. In control animals, BDNF-TdTom signal is visible on the periphery of VE-Cad+ vessels (Fig 1E, left). However, at day 2 following 5 Gy TBI, vascular integrity is markedly disrupted and there is a notable loss of BDNF-expressing cells (Fig 1E, right). Based on the high *TrkB* transcript expression in bone marrow PDGFRα^+^ MSCs, we evaluated whether BDNF treatment induces activation of irradiated human MSCs ex vivo. Primary human MSCs were exposed to 8 Gy irradiation and cultured for 24 hours with or without 100 ng/ml recombinant human BDNF. The cell culture supernatant was screened for differential expression of a panel of 71 human cytokines. Of the 71-cytokines, IL-6 and LIF were significantly increased by BDNF treatment. (Fig 1F).

### Pharmacologic BDNF activation increases bone marrow cellular and structural recovery

**Fig 2:**
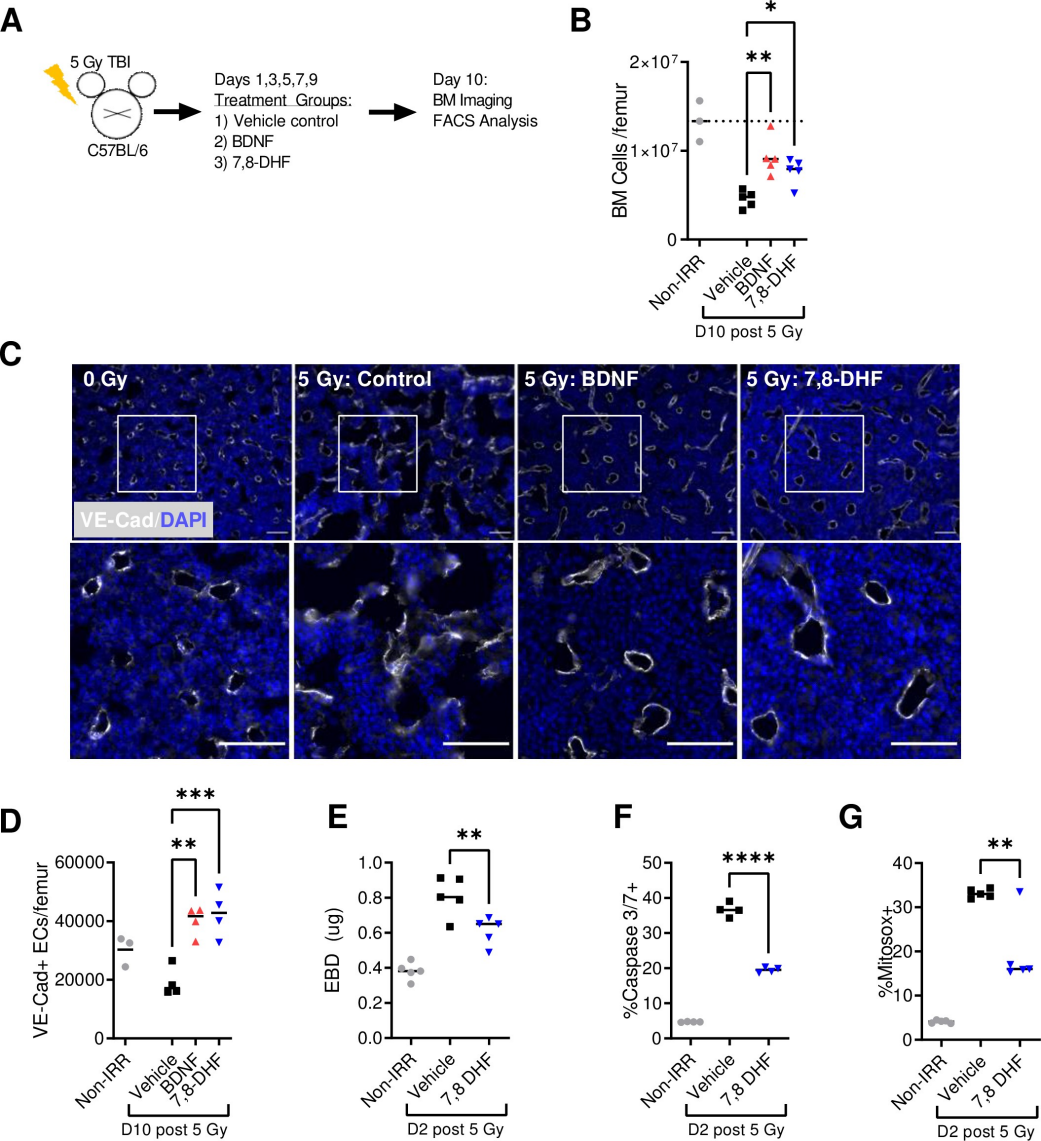
Pharmacologic activation of BDNF-TrkB signaling promotes recovery of the bone marrow niche following radiation injury. (A) Schematic representation of the experimental design: adult C57BL/6 mice were irradiated with 5 Gy and treated starting at 24 hours with vehicle, 0.5 mg/kg BDNF, or 1 mg/kg 7,8-DHF. (B) Total bone marrow cells per femur at day 10 (n = 5 mice per treatment). (C) Representative longitudinal femur sections labeled with VE-cadherin (white) and DAPI (blue) at day 10 following 5 Gy TBI. Scale bar = 100 μm. (D) Total ECs per femur at day 10. (E) EBD permeability at day 2. (F) Percent ECs positive for active caspase 3/7 at day 2. (G) Percent ECs positive for Mitosox at day 2. (n = 4 mice per treatment). *P < .05, **P < .01, ***P < .001, ****P < .0001.

To evaluate if BDNF-TrkB signaling regulates bone marrow recovery following radiation injury, we treated irradiated C57BL/6 mice with either recombinant human BDNF or the small molecule TrkB agonist 7,8-DHF mice (Fig 2A). At day 10 post-injury, irradiated control mice exhibited a loss of total bone marrow cellularity (Fig 2B) and extensive damage to the vascular niche evidenced by immuofluorescent imaging of VE-cadherin labeled blood vessels. Treatment with either recombinant BDNF or 7,8-DHF visibly improved recovery of both total cellularity and vascular structure (Fig 2B, 2C). Total numbers of bone marrow Lin-CD45-VE-Cad+ endothelial cells were calculated by FACS analysis of the contralateral femurs (S1 Fig). Consistent with the observed improvement in vascular structure in Fig 2C, both BDNF and 7,8-DHF treatment signficantly increased total numbers of bone marrow endothelial cells at day 10 after 5 Gy TBI (Fig 2D). At day 2 following 5 Gy TBI and treatment with 7,8-DHF, we assessed functional recovery of vascular integrity by measuring Evans Blue Dye vascular permeability at day 2 post-TBI. Radiation injury acutely increases BMEC permeability; however, agonism of BDNF-TrkB signaling with 7,8-DHF significantly reduces permeability relative to irradiated controls (Fig 2E). At this same time point, 7,8-DHF treatment also significantly reduced endothelial cell apoptosis measured by Caspase 3/7 activation and endothelial mitochondrial reactive oxygen species (ROS) (Fig 2F, 2G).

### Pharmacologic BDNF activation accelerates hematopoietic reconsitution in irradiated mice

**Fig 3:**
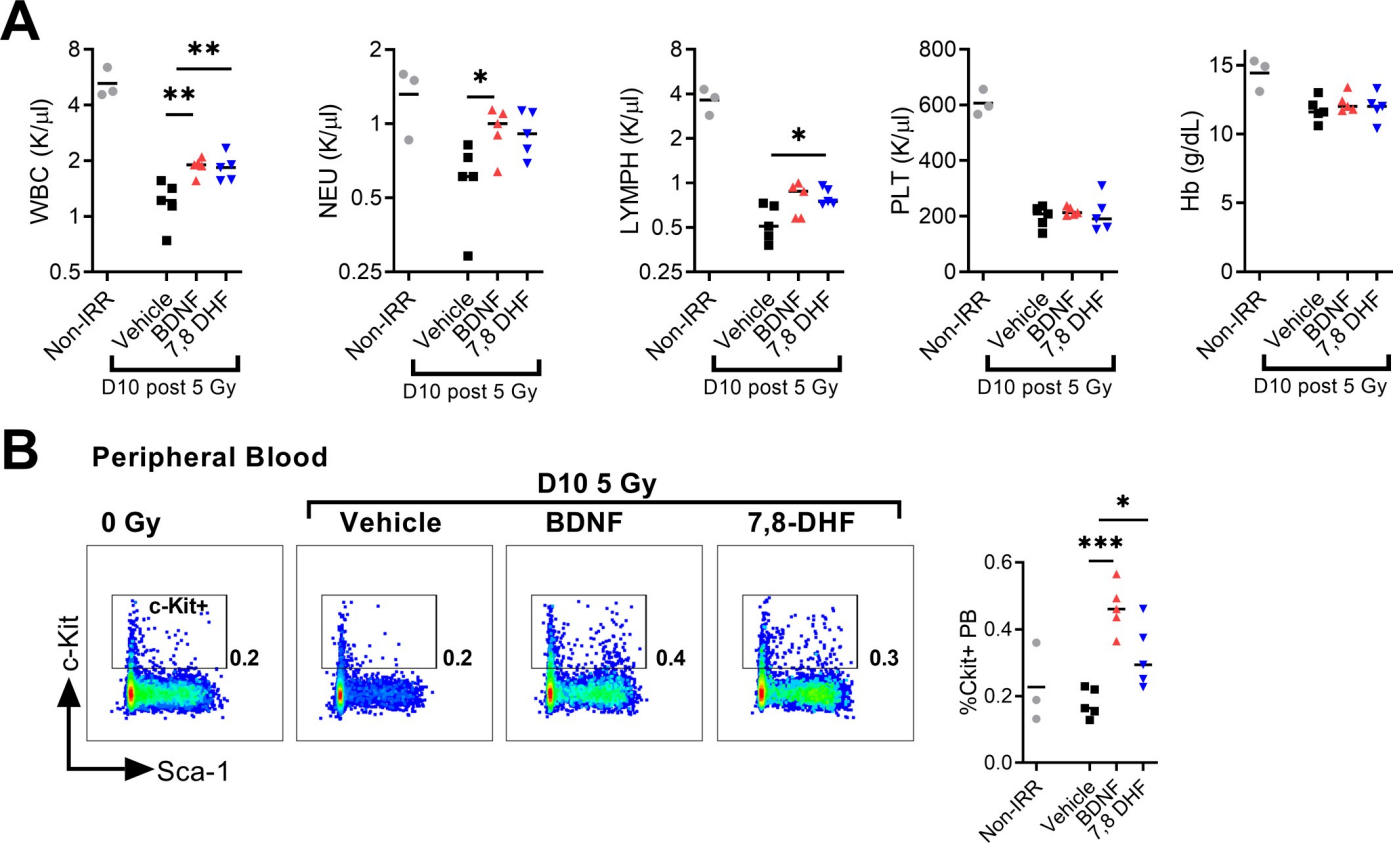
Pharmacologic activation of BDNF-TrkB signaling promotes reconstitution of peripheral blood neutrophils and lymphocytes. (A) Day 10 CBC analysis of total WBCs, neutrophils (NEU), lymphocytes (LYMPH), platelets (PLT), and hemoglobin (Hb). (B) Day 10 peripheral blood analysis of circulating Lin- c-kit+ cells. (n = 5 mice per group). *P < .05, **P < .01, ***P < .001.

Peripheral blood total white blood cell (WBC), neutrophil (NEU), lymphocyte (LYMPH), platelet (PLT) and hemoglobin (Hgb) counts are suppressed in mice receiving 5 Gy at day 10 following TBI (Fig 3A). However, treatment with BDNF significantly increased both total WBC and NEU counts relative to irradiated vehicle controls (Fig 3A). In 7,8-DHF treated mice we observed a similar increase in total WBCs and a significant increase in LYMPH numbers (Fig 3A). Neither treatment had an effect on PLT or Hgb counts. Previously, Kermani et al. showed BDNF treatment following hind-limb ischemia induces recruitment of BM progenitor cells to the site of vascular injury. For this reason, we also evaluated whether BDNF or 7,8-DHF treatment post-TBI promoted mobilization of bone marrow progenitor cells. Here, we observed both BDNF and 7,8-DHF treatment significantly increased mobilization of BM c-kit+ progenitors into the peripheral blood (Fig 3B).

### BDNF treatment promotes recovery of long-term HSCs

**Fig 4:**
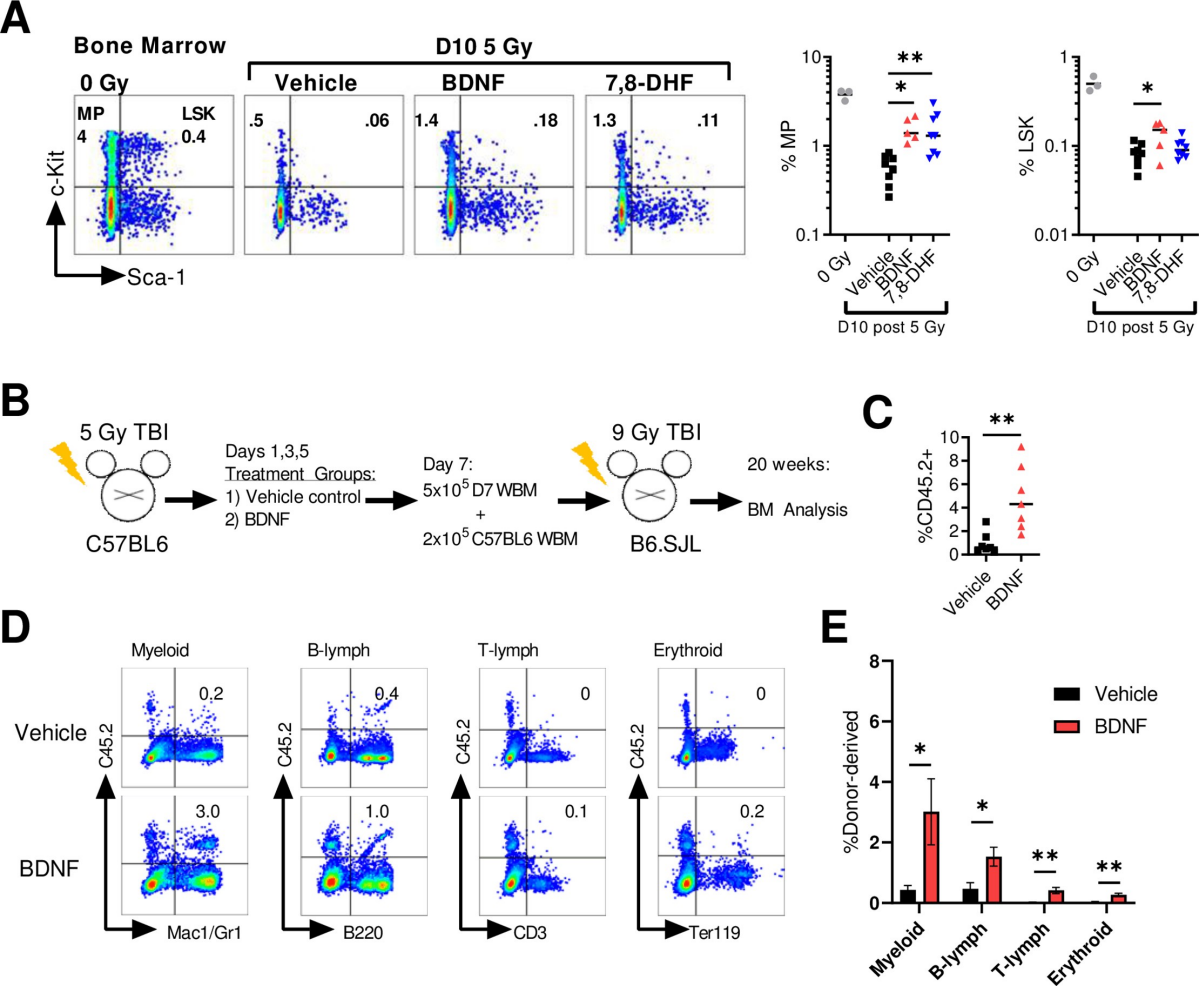
BDNF administration increases bone marrow stem and progenitor cell recovery following TBI. (A) Left, representative day 10 bone marrow FACS analysis of lin-c-kit+ myeloid progenitors (MP) and lin-sca-1+c-kit+ (LSK) cells. Right, %MP and %LSK of the total viable bone marrow cells. (B) Schematic representation of the experimental design: adult C57BL/6 mice were irradiated with 5 Gy and treated starting at 24 hours with vehicle, 0.5 mg/kg BDNF. At day 7, bone marrow was harvested and transplanted competitively into congenic recipient B6.SJL mice. (C) Total bone marrow engraftment at week 20 post-transplant. (D) Representative FACS analysis of bone marrow myeloid, B-lymph, T-lymph, and erythroid engraftment at 20 weeks post-transplant. E, %Donor-derived myeloid, B-lymph, T-lymph, and erythroid engraftment at 20 weeks post-transplant. (n = 7 recipient mice per group). Shown are mean values +/- SEM. *P < .05, **P < .01.

We next evaluated the bone marrow at day 10 following 5 Gy for differences in hematopoietic progenitor and stem cell content. At this dose and time point, radiation injury ablates the myeloid progenitor-enriched population of lineage-c-Kit+Sca-1- cells (MP) as well as the HSC and multipotent progenitor enriched population of lineage-Sca-1+c-Kit+ (LSK) cells (Fig 4A). Treatment with either BDNF or 7,8-DHF significantly increased both the percentage and total number of MP cells (Fig 4A). In the LSK population, BDNF treatment significantly increased the percent and total number of LSK cells (Fig 4A). Finally, we evaluated functional recovery of long-term HSC repopulating function in BDNF treated mice using a competitive transplantation assay (Fig 4B). At twenty weeks post-transplant, bone marrow from BDNF treated mice had significantly higher total donor cell engraftment compared to irradiated controls (Fig 4C). Additionally, BDNF treatment significantly increased multilineage engraftment evidenced by higher myeloid, B-lymph, T-lymph, and erythroid cell engraftment in the BDNF treated group versus control (Fig 4D and 4E).

### BDNF treatment promotes thymic regeneration

**Fig 5:**
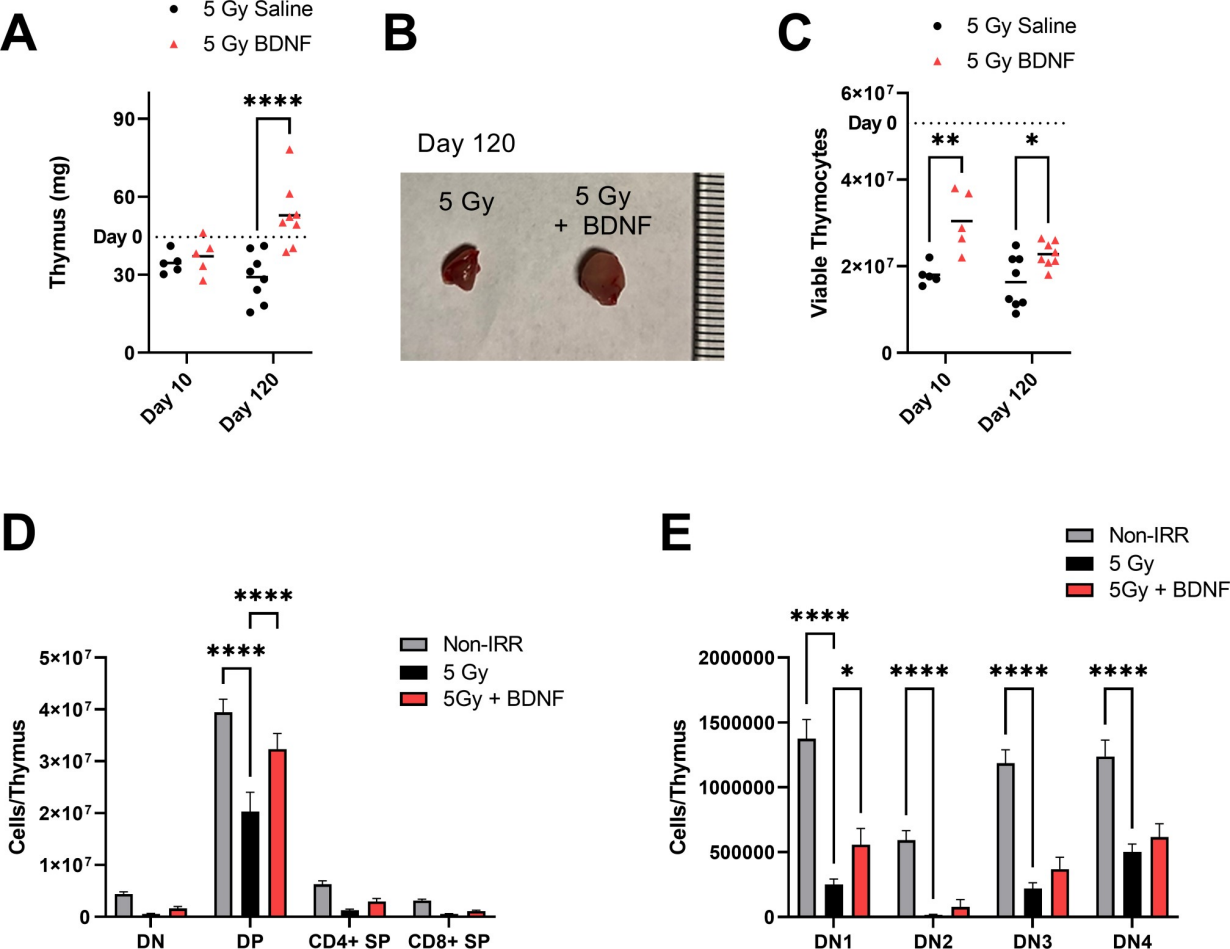
BDNF administration promotes thymic regeneration following TBI. (A) Thymus weights at days 10 and 120 following irradiation and treatment with BDNF. (B) Representative day 120 thymi. (C) Total viable thymocytes at days 10 and 120. (D) Total numbers of double negative (DN), double positive (DP), CD4+ single positive, and CD8+ single positive cells at day 10. E, Total numbers of DN1, DN2, DN3, and DN4 thymocyte precursors at day 10. (Day 10: n = 5 mice per group). Shown are mean values + SEM. *P < .05, **P < .01, ****P < .0001.

Since BDNF and its receptor TrkB are expressed in the thymus, we next evaluated whether exogenous BDNF administration following radiation injury regulated both early (day 10) and late (day 120) recovery of the thymus. At day 10 following radiation, there is a decrease in total thymus mass compared to day 0 non-irradiated controls (dotted line) in both the saline and BDNF treated groups (Fig 5A). However, at 120 days post-injury, the thymus is significanltly enlarged in the BDNF treated group compared to irradiated controls (Fig 5A and 5B). It is notable that the irradiated BDNF treated animals had larger thymi at day 120 compared to the day 0 control as the thymus typically undergoes involution with age. Treatment with BDNF also increased recovery of total viable thymocytes at both days 10 and 120 post-injury compared to irradiated controls (Fig 5C). At day 10 post-injury, we analyzed thymocyte subsets to evaluate whether BDNF treatment promotes recovery of specific thymocyte populations following injury (Fig 5D, S2 Fig). Double positive (DP) thymocytes expressing both CD4 and CD8 are the population most decreased at day 10 following radiation injury. Treatment with BDNF significantly improves recovery of DP thymocytes (Fig 5D). Additionally, within the double negative (DN) thymocytes, radiation significantly decreases all subsets of thymocytes. Treatment with BDNF significantly increases the DN1 thymocytes (Fig 5E).

## Discussion

Labyourie et al. first characterized expression of neurotrophins and the Trk family receptors in adult and fetal human bone marrow where they found transcript levels of the four members of the neurotrophin family (nerve growth factor, BDNF, neutrophin-3, and neutrophin-4) were increased in the fetal bone marrow compared to adult bone marrow [17]. These data suggest neurotrophins may play a key role in early hematopoietic development. Additionally, Labyourie et al. performed immunological detection of neurotrophin receptors and noted TrkB positive staining in blood vessel associated adventitial reticular cells [17]. More recently, Rezaee et al. demonstrated human bone marrow stromal cells express TrkB and respond to BDNF treatment by increasing levels of IL-6 cytokine production [28]. Primary human bone marrow endothelial cells have also been documented to express TrkB and have an increased angiogenic response to in vitro BDNF treatment [29]. Here, we have characterized BDNF and TrkB expression in the spleens, thymus, and bone marrow of pediatric and adult mice. Consistent with the early work by Layourie, we do see a decrease in bone marrow endothelial cell *Bdnf* transcript levels with increasing age. Additionally, we observed dynamic regulation of *TrkB* transcript expression with age in the thymus and bone marrow.

Our prior data demonstrated endothelial progenitor cells expressing high levels of BDNF promote hematopoietic reconstitution following ablative radiation injury *in vivo* [2]. Based on this work, we hypothesized BDNF signaling may be mediating part the regenerative effect observed with endothelial progenitor cell infusion. The time-dependent loss of endogenous pro-BDNF in the bone marrow following radiation injury would suggest endogenous BDNF production in the bone marrow plays a role in maintaining homeostasis in the bone marrow microenvironment. Therefore, we evaluated whether exogenous administration of either BDNF or the TrkB agonist 7,8-DHF promoted bone marrow recovery following radiation injury. Here, we observed that systemic administration of either BDNF or TrkB agonist 7,8-DHF accelerated the recovery of bone marrow function and increased hematopoietic stem cell content. In the vascular niche, we observed treatment with 7,8-DHF significantly reduced radiation-induced endothelial injury at 48 hours as evidenced by reductions in mitochondrial ROS, apoptosis, and permeability. By day 10, treatment with either BDNF or 7,8-DHF significantly increased total numbers of bone marrow endothelial and hematopoietic cells and accelerated reconstitution of mature immune cells. Additionally, BDNF treatment promoted recovery of a hematopoietic stem cell population capable of multi-lineage reconstitution. These data suggest BDNF-TrkB signaling in the bone marrow may be important regulator of hematopoietic reconstitution in vivo following radiation injury.

Additionally, we have seen that BDNF administration in vivo markedly increases the regeneration of the thymus following radiation injury. BDNF protein levels are known to decline in the thymus with age [30]. Prior studies have shown that BDNF is produced by the thymic stroma and acts on TrkB+ thymocytes [21, 27, 30, 31]. TrkB deficiency in the thymus leads to apoptosis of thymic lymphocytes [21] and treatment with BDNF has been shown to promote survival of thymocyte precursors in vitro [31]. Additionally, in vivo studies with BDNF deficient mice have demonstrated BDNF regulate thymocyte maturation at the DN3 to DN4 stage [27]. In the context of radiation injury, we have observed that BDNF promotes thymocyte survival and supports a long-term recovery of thymic function as we see increased numbers of thymocytes at day 120 following injury.

Since agonism of BDNF-TrkB signaling supports regeneration of both the bone marrow and thymus following radiation injury, these data would support the therapeutic use of BDNF or 7,8-DHF for the treatment of radiation-induced neutropenia and lymphocytopenia. However, it should be noted that BDNF and TrkB upregulation are associated with tumor cell survival in a number of solid cancers [32, 33] and in acute leukemias [34]. We have not observed the development of tumors in our long-term cohorts of mice treated with BDNF or 7,8-DHF (120 days following TBI). We do not expect to observe malignancies in these cohorts as long-term 7,8-DHF treatment has been shown to be safe and non-oncogenic in preclinical animal models of traumatic brain injury, Parkinson’s disease, Alzheimer’s disease, Huntington’s disease, and stroke [5–10].

Prior publications have demonstrated TrkB is expressed on human CD34+ cells (16, 17), murine B-lympocytes (18), as well as murine thymocytes (19). However, in our transcriptional analysis of bone marrow hematopoietic stem and progenitor cell populations, we did not detect *TrkB* expression. Therefore, it is unlikely that exogenous BDNF administration is mitigating radiation injury to the bone marrow by acting directly on the hematopoieitic stem or progenitor cell compartment. Instead, our data indicate BDNF acts on TrkB+ bone marrow MSCs to promote the release of regenerative hematopoietic cytokines. This finding is supported by data from a recent lung injury study by Paris et al. which demonstrated BDNF promoted alveolar regeneration via activation of TrkB expressing pulmonary mesenchymal cells [16]. MSCs have been used as a vehicle to deliver BDNF as well as other neurotrophic factors for treatment of neurological and ischemic injury [35]. In these studies with BDNF overexpressing cell lines, there is evidence to suggest BDNF may also be acting in an autocrine to enhance MSC production of trophic factors [36]. This is consistent with the observed ability of transplanted MSCs to functionally respond to the local immune microenvironment by altering cytokine secretion [37, 38]. Here, we observed BDNF stimulates the secretion of IL-6 and LIF from irradiated bone marrow MSCs. These two factors have previously been shown to promote bone marrow reconstitution following radiation injury. In irradiated mice, IL-6 has been shown to stimulate multilineage hematopoietic reconstitution [39] and both IL-6 and LIF improve platelet recovery in irradiated rhesus monkeys [40, 41].

Our data with the TrkB specific agonist 7,8-DHF would suggest the regenerative activity of BDNF in the bone marrow is mediated primarily via activation of TrkB. However, BDNF is also known to bind and activate the low-affinity nerve growth factor receptor (p75NTR or NGFR). Recently, Severe et al. have reported that bone marrow stromal cell fractions expressing NGFR are resistant to radiation injury and express high levels of SDF1 [42]. Therefore, it is possible that BDNF activity may be mediated by activation of this distinct NGFR+ population of stromal cells. In the future, we plan to discriminate which subsets of mesenchymal cells respond to BDNF treatment within the bone marrow microenvironment using Cre-LoxP mouse models to conditionally delete TrkB or NGFR from MSC cell populations.

## Conclusion

In conclusion, our data support a novel role of BDNF in regulating bone marrow regeneration and hematologic reconstitution following myelosuppressive radiation injury through the TrkB receptor. Pharmacologic treatment with BDNF or TrkB receptor agonist may have therapeutic benefit for treatment of radiation or chemotherapy induced myelosuppression.

## Supporting information

S1 Fig Left, representative FACS gating strategy for VE-Cad+ EC analysis in the bone marrow. Right, %Lin-VE-Cad+ ECs.
(PDF)

S2 Fig Left, representative FACS gating for the following thymocyte populations: Double negative (DN), double positive (DP), CD4+ single positive, and CD8+ single positive. Right, analysis of thymocyte precursor subsets within the DN population: DN1, DN2, DN3, and DN4 based on CD44 and CD25 staining.
(PDF)
