# Supplementary figures and images for: Brain-derived neurotrophic factor promotes immune reconstitution following radiation injury via activation of bone marrow mesenchymal stem cells
Source: PLoS One. 2021 Oct 25;16(10):e0259042. doi: 10.1371/journal.pone.0259042 (PMC8544859; doi:10.1371/journal.pone.0259042)

Supp Fig 1 **0 Gy**

**5 Gy: Control**

**5 Gy: BDNF**

**5 Gy: 7,8-DHF**

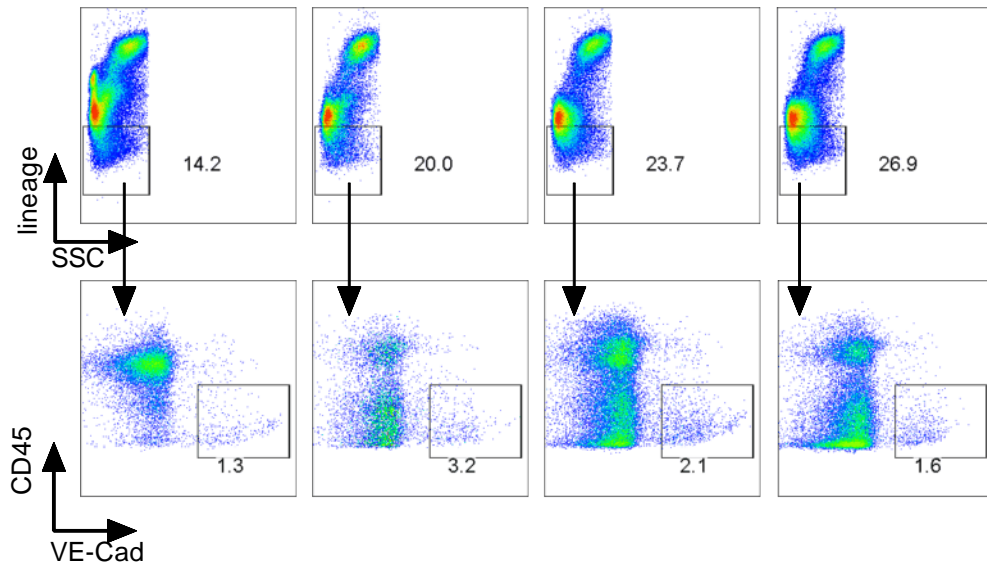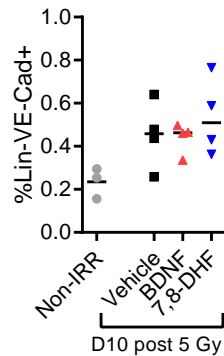

Supplement: S1 Fig — Right, %Lin-VE-Cad+ ECs. (PDF) [file pone.0259042.s001.pdf]

Supp Fig 2

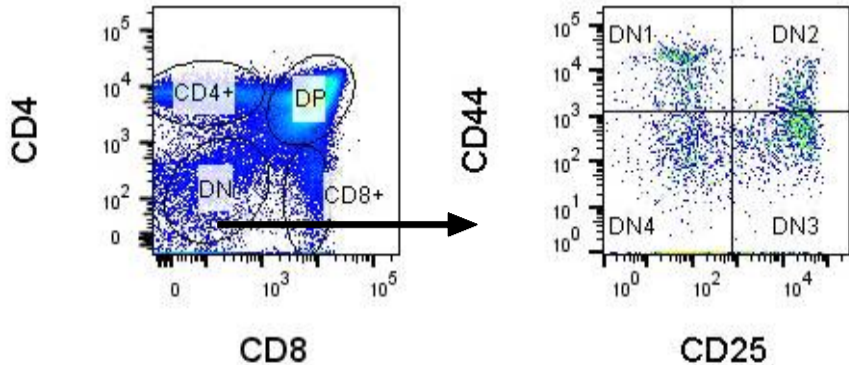

Supplement: S2 Fig — Right, analysis of thymocyte precursor subsets within the DN population: DN1, DN2, DN3, and DN4 based on CD44 and CD25 staining. (PDF) [file pone.0259042.s002.pdf]
